# Blueprint for Large-Scale Silicon Optical Phased Array Using Electro-Optical Micro-Ring Pixels

**DOI:** 10.1038/s41598-017-18040-3

**Published:** 2017-12-18

**Authors:** Che Zhao, Chao Peng, Weiwei Hu

**Affiliations:** State Key Laboratory of Advanced Optical Communication Systems and Networks, School of Electronics Engineering and Computer Science, Peking University, Beijing, 100871 China

## Abstract

We propose a modularized architecture of a large-scale optical phased array (OPA) on a silicon on insulator (SOI) platform, using electro-optical (EO) pixels. Each pixel contains a directional coupler, a micro-ring phase shifter, and a grating optical antenna, on a compact configuration of area 50 *μ*m × 50 *μ*m, with optical and electrical interconnections. Moreover, we present an exemplary blueprint of an OPA consisting of 32 × 32 EO pixels, which sets the width of the main lobe as 0.04° × 0.04° and the field of view as 1.78°. By applying an over-coupled condition, the modulation efficiency and the accompanying intensity modulation are balanced, thus, the OPA performance is not severely degraded. The discussion on the fabrication tolerance shows that the proposed architecture is robust and feasible regarding the state-of-the-art fabrication process, and the performance of the main lobe width and field of view can be further optimized by a larger system size and smaller pixel size. Furthermore, the complexity of interconnections linearly depends on the number of rows and columns, making it highly scalable.

## Introduction

Over the past decades, optical phased arrays (OPAs) have attracted significant attention owing to their promising capabilities of non-inertial optical beam forming and steering. By manipulating the specific spatial distribution of the amplitudes and phases emitted from individual optical antennas, OPAs can form the desired radiation patterns through far-field interference, which enables numerous applications, such as laser radar (Lidar), 3D imaging, display, and optical communications^1–7^. Several types of OPAs, such as laser arrays^8–12^, liquid crystals^13–18^, as well as micro-electro-mechanical systems (MEMS)^19–24^, have been proposed and demonstrated. However, the application of these OPAs is limited by challenges of large-scale integration or high-speed operation, which are crucially important for several applications.

The recent development of silicon photonics provides a new approach to realizing OPA systems. A large-scale OPA with thousands or an even greater number of pixels can be integrated on a single, compact, and low-cost chip. Besides, its fabrication process is fully compatible with the conventional complementary metal–oxide–semiconductor (CMOS) technologies, making it suitable for mass production. Several prototypes of silicon OPAs have been reported, and an OPA consisting of 64 × 64 pixels has been demonstrated^25^.

Phase manipulation is one of the key functions for realizing an integrated silicon OPA. Different mechanisms, such as thermo-optical (TO) modulation^25–31^, MEMS^19–24^, as well as electro-optical (EO) modulation^32^ have been adapted to control the phases of the emitted beams. Among these, EO modulation has been considered as the most promising choice owing to the significant advantage of its response time^33,34^. As the typical response time of MEMS and TO modulation is only approximately several microseconds (~*μ*s)^19,35^, EO modulation can be more competitive for high-speed applications.

The main drawback of EO modulation comes from its low modulation efficiency. Due to the poor efficiency of the plasma dispersion effect, EO phase shifters are quite sizeable (with waveguide lengths up to hundreds of *μm*). As a result, a silicon OPA must be divided into different regions for phase shifters and optical antennas; hence, the complexity of the optical and electronic interconnections exponentially grows with the size of the OPA. Alternatively, a modularized design has been proposed to reduce the complexity of interconnections. Two main functional parts, specifically a TO modulation based phase shifter and an optical antenna, are integrated as a “pixel” to construct an OPA^25,35,36^. A waveguide bus has also been adapted to distribute the light, and a series of row/column control wires is used as the fabric of electronic interconnections.

The modularized design has shown considerable advantages and potential in realizing large-scale silicon OPAs. Although TO modulation is more efficient than EO modulation, its low modulation speed is its main drawback. In this work, we propose a modularized blueprint for a large-scale silicon OPA, by using micro-ring phase shifters as EO modulation pixels. Owing to the enhancement of optical resonance within the micro-rings, such a phase shifter can achieve high modulation efficiency, compact footprint, and maintain high modulation speed at the same time. We propose a pixel design consisting of a directional coupler, EO-based micro-ring phase shifter, and an emitting optical antenna. A waveguide distributed network and an electronic control fabric are adapted to realize a suitable complexity of the interconnections.

Owing to the improvement of the modulation efficiency, introduced by the optical resonance, sufficient phase changes can be achieved in an integrated “pixel” with small footprint, and as a result, the OPA can be “modularized”. However, the sharp optical resonance also causes unwanted intensity modulation, specifically, different phase shifts are accompanied by non-uniform insertion losses, which can degrade the performance of the OPA. Therefore, an accurate balance must be found between the modulation efficiency and the accompanied intensity modulation. Nevertheless, as optical resonances are susceptible to the imperfections in fabrication, the resonance wavelengths, as well as the Q factors of the micro-rings, shift and fluctuate due to the fabrication faults bringing more challenges to the fabrication process.

In this work, we present a two-dimensional (2D) OPA with a size of 32 × 32 pixels, in which the EO micro-ring pixels are suitably designed to achieve balanced performance in terms of modulation efficiency, intensity uniformity, and fabrication robustness. The micro-ring works in an over-coupled condition with a Q factor of ~2700 with an evanescent coupling gap of 150 nm. We proved that the accompanying intensity modulation does not degrade severely the performance of the OPA. Confirmed by a series Monte Carlo simulations, the device is robust and feasible with respect to the fabrication precision required by the state of the art silicon photonics foundries.

The remainder of this paper is organized as follows. In Section *System architecture and key components* we present the architecture of the modularized silicon OPA with EO micro-ring pixels, and analyze the characteristics of the key individual components, respectively. In Section *System performance and tolerance analysis* we discuss the performance of a 2D OPA with 32 × 32 pixels, as well as its tolerance to the fabrication imperfections. In Section *Section Conclusion*, we conclude our findings.

## System architecture and key components

**Figure 1:**
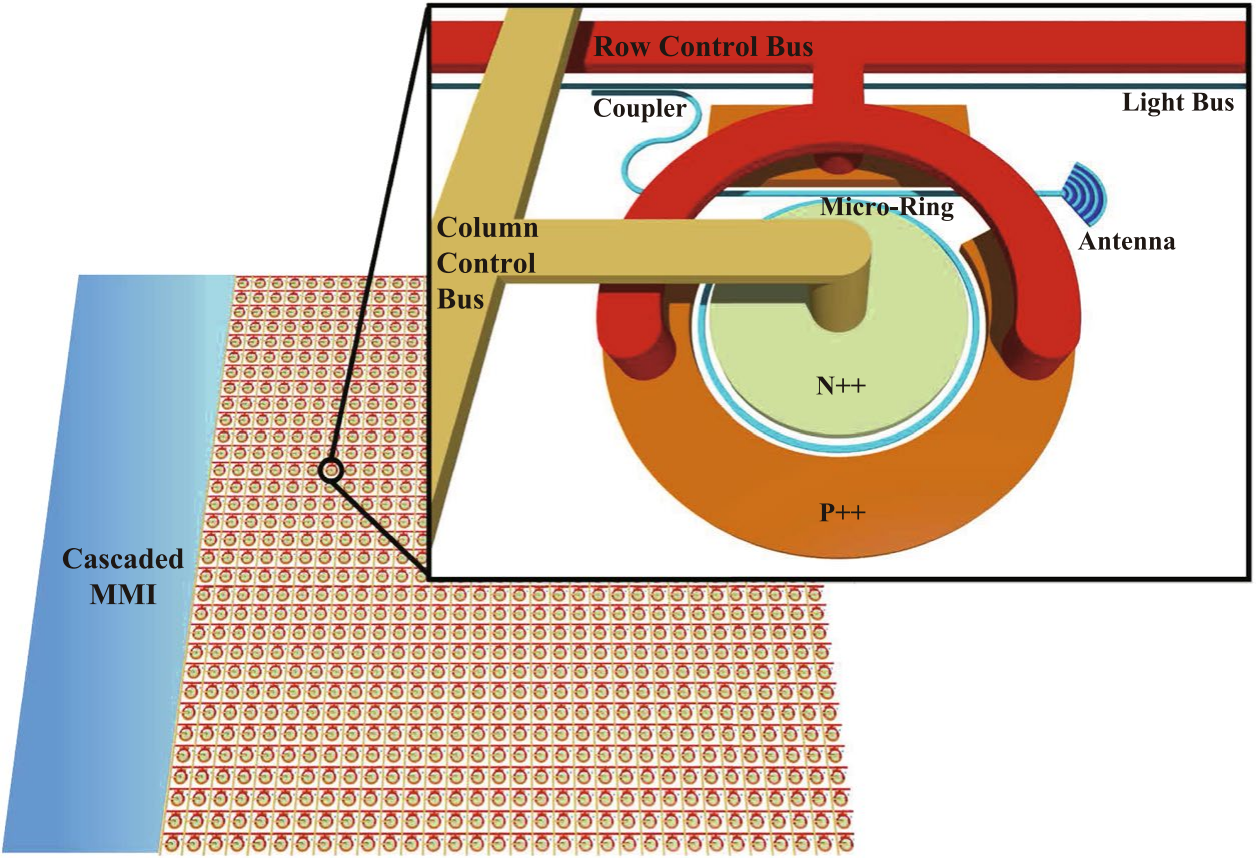
Schematic of the silicon OPA architecture consisting of a series of modularized, EO modulation based micro-ring pixels; a set of light buses for distributing the incident light; and a set of control buses for electronically manipulating the optical phases.

The proposed modularized architecture of the silicon OPA system is schematically illustrated in Fig. 1. The incident light generated by a coherent laser source is split by a cascade multi-mode interferometer (MMI), and then coupled into several waveguides, operating as “light buses” to distribute the light into the individual OPA units or “pixels”. Each pixel consists of three functional parts, including a directional coupler, which drops the incident light by evanescent coupling; a micro-ring phase shifter, using the plasma dispersion effect to manipulate the phases of the light beam; and an arc-shaped optical antenna, which emits the light into free space. This modularized EO pixel is the fundamental building block in the construction of the large-scale silicon OPA system. A set of column and row metal wires is connected to the *N* ^++^ and *P* ^++^ contacts of the individual micro-ring phase shifters, which work as addressable “control buses” to manipulate the voltages applied on each micro-ring. By incorporating a large number of the EO pixels, the sophisticated far-field patterns desired can be achieved by the interference of the emissions from the individual optical antennas.

The proposed architecture is modularized, as all functional parts have been integrated into a single pixel. Consequently, such an OPA system does not need to be divided into separate modulation region and emission region, which can significantly reduce the complexity of the optical and electronic interconnections. In the following parts of this section, we present further details of these key components in the proposed OPA system.

### Directional coupler

**Figure 2:**
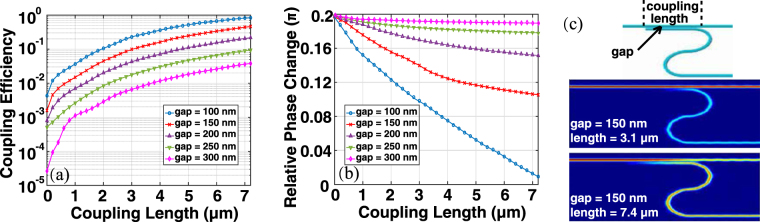
Coupling efficiency (**a**) and relative phase shift (**b**) with different coupling lengths and gaps. (**c**) Lengths of 3.1 *μ*m and 7.4 *μ*m under a gap of 150 nm, giving coupling efficiencies of 10% (−10 dB) and 50% (−3 dB).

In every EO pixel, a directional coupler is employed to evanescently drop the incident light from the light bus into the micro-ring. Considering the state of the art precision of the fabrication process, we investigate the gaps from 100 nm to 300 nm around *λ* = 1550 nm. As shown in Fig. 2(a), the coupling efficiency can be tuned by the length and gap of the coupling region, which sets the micro-ring to be either over-coupled or critical-coupled. As discussed in the following section, the Q factor can be remarkably high when the micro-ring and coupler working under critical-coupling condition, which can exhibit a serious problem for the OPA because it causes an unacceptable uniformity in the intensity with respect to the different phase modulations. In this work, we found that a gap of 150 nm can be optimal to make the micro-ring working under the over-coupled condition.

Tuning the coupling length provides a freedom to compensate the losses during light distribution, in order to achieve the desired emission power at every EO pixel. As the pixels located differently in the light distribute network, their overall propagating losses (including the absorption and scattering losses) are not identical. For instance, a shorter coupling length can be chosen for the pixels, which are closer to the light source comparing to those, which are far away. Moreover, scatter losses, due to the imperfections of the fabrication, can also be compensated by tuning the coupling length during the adaptive and recursive optimizations of the fabrication process. However, as illustrated in Fig. 2(b), the relative phase shifts brought by different coupling lengths may cause problems to such tunability. For a varying coupling length, the phase shift must be as flat as possible. As can be seen in Fig. 2(b), from this perspective a gap of 150 nm also gives acceptable performance.

In particular, we present two typical coupling lengths at a gap of 150 nm (the blue insets in Fig. 2(c)): the lengths of 3.1 *μ*m and 7.4 *μ*m give coupling efficiencies of 10% (−10 dB) and 50% (−3 dB), respectively, and the relative phase differences between them are less than 0.05*π*. Besides, according to our experiments, the loss of the S-band bending is under 0.6 dB.

It should be noted that, certain well-designed, non-uniform distributions of the emission powers can benefit from the suppression of the side lobes^37^. Tuning the coupling length can be an effective approach to distribute these non-uniform emission powers. As discussed in Section *System performance and tolerance analysis*, the non-uniform power distribution improves the OPA performance of side-lobe suppression ratio performance.

### Micro-ring phase shifter

The micro-ring phase shifter is the most critical component in the EO pixel. By using the plasma dispersion effect, the EO modulation based micro-ring has been considered to be both high-speed and high-efficiency^38,39^. To be compatible with the standard semiconductor process, we design an all-pass ring resonator on a silicon on insulator (SOI) wafer with a silicon layer thickness of 220 nm, and choose the width and height of the waveguide rib to be 450 nm, and 130 nm, respectively, and the radius *R* to be 10 *μ*m. The EO modulation region can be realized by doping boron and phosphorus elements in intrinsic silicon. The cross section is a typical transverse p-i-n structure of the ridge waveguide. The optical cavity in the middle is made by intrinsic silicon, and the active regions are doped with boron (*P* ^++^ doping, 10^20^/cm^3^) and phosphorus (*N* ^++^ doping, 10^20^/cm^3^), respectively. Besides, two *P* ^++^ regions are placed around the ring, in order to improve the modulation efficiency.

When a forward voltage is applied, the majority of carriers on both sides are injected into the intermediate intrinsic region, which leads to a change in the effective refractive index of the intrinsic region. The modulation of the refractive index is achieved by the carrier transportation, and as a result, it determines the modulation efficiency. The response speed is dominated by the carrier recombination time, which is typically within 10 ns. However, owing to the high quality factor of the optical cavity, a large enough phase shift can be achieved when the carrier injection has not yet reached the steady state, thus, the system response time can be further reduced to the scale of 1 ns or even less^40^.

**Figure 3:**
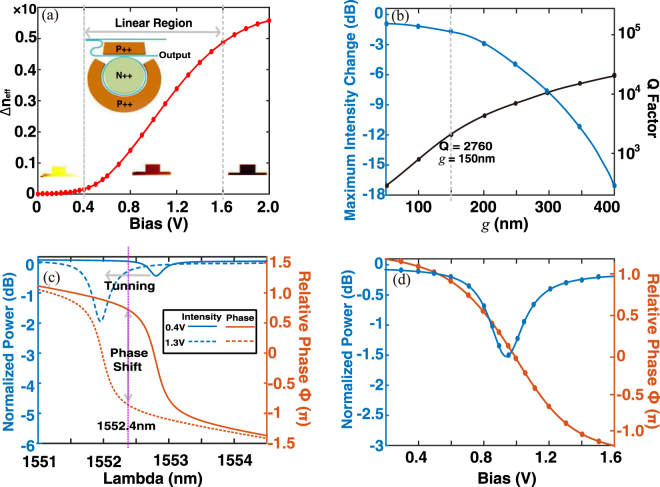
(**a**) Change of effective refractive index vs. bias voltage. (**b**) Change of maximum output intensity under different coupling gaps *g*. (**c**) The tuned resonance wavelength and the obtained amplitude and phase response for the coupling gap *g* = 150 nm. (**d**) Detailed intensity changes and phase shifts vs. bias voltage under *g* = 150 nm and *λ* = 1552.4 nm.

We present numerical simulations by using finite-difference time-domain (FDTD) and finite element method (FEM) algorithms, to investigate and optimize the optical and electrical characteristics of the EO micro-ring. Firstly, the optical modal patterns were calculated by using FDTD Solutions according to the structural and material parameters of the micro-ring, while the carrier transportation properties were calculated by Silvaco Atlas to obtain the carrier density distribution under a given voltage bias. Further, the change in refractive index induced by plasma dispersion is given by the Drude–Lorenz model at *λ* = 1550 nm, as^33^:

1 $${\rm{\Delta }}n={\rm{\Delta }}{n}_{e}+{\rm{\Delta }}{n}_{h}=-\mathrm{[8.8}\times {10}^{-22}\,{\rm{\Delta }}{N}_{e}+8.5\times {10}^{-18}\,{({\rm{\Delta }}{N}_{h})}^{0.8}]$$

2 $${\rm{\Delta }}\alpha ={\rm{\Delta }}{\alpha }_{e}+{\rm{\Delta }}{\alpha }_{h}=8.5\times {10}^{-18}\,{\rm{\Delta }}{N}_{e}+6.0\times {10}^{-18}\,{\rm{\Delta }}{N}_{h}$$

where Δ*N*_*e*_, Δ*N*_*h*_ are the density modulation of electrons and holes, respectively. Thus, the phase and amplitude response can be calculated accordingly. As the carrier injection brings perturbations to the optical modal field, such calculation procedure can be performed in a recursive manner to achieve sufficient accuracy. As presented in Fig. 3(a), for a given voltage bias from 0.4 V to 1.6 V (linear region marked by gray dashes), the effective refractive index of micro-ring is linearly modulated in about 5 × 10^−3^/100 mV. By increasing the voltage bias further, less effective modulation occurs due to the saturation of the carrier injection^41^.

It should be noted that, the intensity and phase response of the micro-ring are simultaneously modulated when its resonance peak shifts. The normalized intensity *I* and phase shift *ϕ* can be written as

3 $$\begin{array}{ll}I=\frac{{a}^{2}-2ra\,{\cos }(\theta )+{r}^{2}}{1-2ar\,{\cos }(\theta )+{(ra)}^{2}}, & \varphi =\frac{{a}^{2}-2ra\,{\cos }(\theta )+{r}^{2}}{1-2ar\,{\cos }(\theta )+{(ra)}^{2}}\end{array}$$

where *θ* = *βL*, with *β* being the propagation constant of micro-ring waveguide and *L* is the round trip length. *a* is the amplitude transmission coefficient with *a* ^2^ = *e* ^−*αL*^ and *α* is the attenuation coefficient. The self-coupling coefficient *r* and cross-coupling coefficient *κ* follow *r* ^2^ + *κ* ^2^ = 1 assuming that there are no losses in the coupling region^42^. As the micro-ring works as a phase shifter, the accompanying intensity modulation is actually equivalent to those extra insertion losses, which are not identical with respect to the different phase shifts. As a result, the micro-ring has to work in an over-coupled regime (*r* < *a*) to obtain a nearly flat response, which can minimize the effect of the unwanted intensity changes. The maximum intensity change and related Q factors for different coupling gaps *g* were calculated by simulations, as shown in Fig. 3(b). In general, for the over-coupled regime, a larger *g* brings stronger coupling, resulting in a higher Q factor and modulation efficiency. However, at the same time, the change in intensity is more significant and can severely degrade the OPA performance. An accurate balance between the modulation efficient and intensity modulation has to be found.

Considering the results above, we present a specific implementation at *g* = 150 nm. In this case, voltage bias values of 0.4–1.3 V give a nearly full 2*π* phase shift with intensity changes of only 1.5 dB, the corresponding Q is approximately 2760 (assuming a waveguide loss of 3 dB/cm), *r* is ~0.85, and *a* is ~0.96. (Fig. 3(c)). For a given incident wavelength *λ* = 1552.4 nm (purple dash in Fig. 3(c)), the detailed intensity and phase responses are provided in Fig. 3(d). The maximum intensity change is approximately 1.5 dB, which appears at the resonance peak. As discussed in a later section, such an EO micro-ring structure can provide a satisfactory performance for OPA systems.

### Optical antenna

The light is emitted from an optical antenna in the EO pixel. For an on-chip OPA system, a compact footprint is expected for an integrated optical antenna. Generally, grating couplers are used for the light emission with a length of typically a few dozen *μ*m^43^. These are still too sizeable to be integrated into the EO pixel and also exhibit difficulties for the construction of 2D arrays. Here, we employ an arc-shaped grating coupler as the optical antenna, similar to those used by Sun. *et al*.^25,36^.

**Figure 4:**
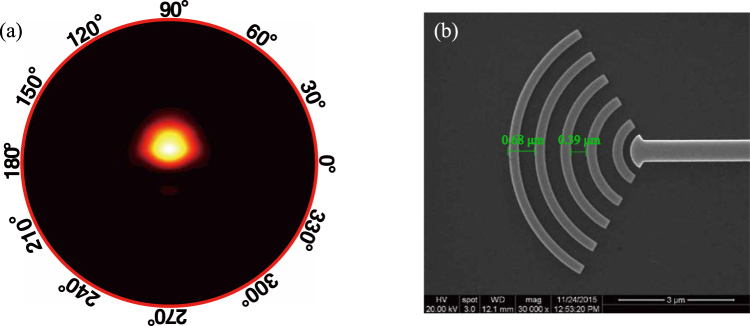
(**a**) Far-field radiation pattern of the proposed arch-shape grating coupler as optical antenna. (**b**) SEM image.

An arch-shaped grating coupler is compact and easy to fabricate. We optimized its structural parameters for our purpose. The length of the arc-shaped antenna is 3.5 *μ*m and its width is 6.4 *μ*m. The period of the two adjacent arcs is 0.68 *μ*m and the gap between them is 0.39 *μ*m. Numerical simulations suggests that the antenna has 50° × 40° diffraction angles in its far-field pattern (Fig. 4(a)). A scanning electron microscope (SEM) image is shown in Fig. 4(b). The arc-shaped antenna exhibits an excellent performance in the C-band. Our experimental results verify that the antenna has an insertion loss of only 4.5 dB to 6 dB around 1550 nm and has high fabrication tolerance.

### Interconnections

One of the main challenges in the realization of a large-scale integrated OPA comes from the difficulties of the interconnections. In a conventional OPA architecture, the system has to be divided into separated regions for the modulation and emission, mainly due to the sizeable dimensions of the phase shifter. As a result, the complexity of the interconnection exponentially increases with the size of the OPA, thus, the routing and layout of the optical and electrical paths become difficult.

In our proposed scheme, the light is distributed by backbone waveguides, referred to as “light bus”, and then dropped into each pixel by the directional coupler. For an OPA with *N* × *N* pixels, only *N* light buses are required. Such degree of light splitting is feasible by adapting a cascade MMI or Y-branches light splitter.

To deliver the modulation signals to each of the pixels, an electrical interconnection fabric is required. As shown in Fig. 1, we used a “control-bus” structure to support the electrical interconnection. The row control buses (red lines) are contacted with the *P* ^++^ region and the column control buses (yellow lines) are contacted with the *N* ^++^ region. Electric signals are applied to tune the voltages between the rows and columns, which act on each of the EO pixels. When a certain voltage bias is applied between the *P* ^++^ and *N* ^++^ regions, it shifts the resonance wavelength of the micro-ring, leading to an effective phase response.

## System performance and tolerance analysis

### System design and performance

Based on the proposed modularized OPA architecture, we present an implementation of an on-chip OPA consisting of 32 × 32 = 1024 electro-optical pixels. Each pixel has a 50 × 50 *μ*m footprint, and the overall size of the OPA device is about 1.6 mm × 1.6 mm. The device can be fabricated in a commercial photonic foundry by using a standard CMOS process.

The far-field intensity distribution is determined by the interference of the emissions from the individual OPA pixels, which can be written as^1^:

4 $$\begin{array}{ll}I=\frac{{{\sin }}^{2}(N\delta )}{{N}^{2}\times {{\sin }}^{2}(\delta )}, & {\rm{with}}\,\delta =\frac{\pi d\,{\sin }\,\psi }{\lambda }-\frac{\phi }{2}\end{array}$$

where *λ* is the free-space wavelength; *ψ* is the field angle (relative to the boresight of the array); *N* is the number of pixels in the array; *d* is the spacing between the adjacent optical antennas, assuming a uniform phase difference *φ*. According to Eq. 4, the width of the main lobe in the proposed OPA is only 0.04° × 0.04°, with a field of view of 1.78°.

As mentioned earlier, the coupling length provides a freedom of tuning the power of light, which evanescently couples into each pixel. As a result, a non-uniform power distribution^37^ can be realized to improve the side-lobe suppression ratio of the beam emission. Here, we present a series of coupling lengths (listed in Table 1) for the proposed 32 × 32 system. For a state-of-the-art fabrication process, the precision of the length is set to 10 nm and the power fluctuation is under 0.2 dB, correspondingly. According to the results in Fig. 2(b), the relative phase shifts between the individual pixels are less than 0.05*π*.

**Table 1:** Coupling lengths for the non-uniform power distribution.

Number of Coupler	Coupling Lengths (*μ*m)							
1–8	0.22	0.54	1.01	1.38	1.77	2.14	2.52	2.82
9–16	3.16	3.56	3.91	4.21	4.47	4.73	4.99	5.22
17–24	5.40	5.54	5.64	5.72	5.73	5.69	5.61	5.46
25–32	5.28	5.06	4.79	4.48	4.16	3.83	3.61	3.69

**Figure 5:**
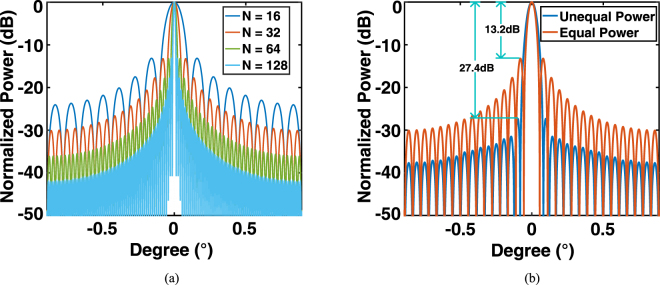
Far-field angular distribution of the beam emission. (**a**) For system sizes of *N* × *N*, where *N* = 16, 32, 64, and 128, respectively. (**b**) For the uniform and non-uniform power distributions in the system of a size of 32 × 32, the marks show the improvement of the side-lobe suppression ratio.

The performance of the non-uniform power distribution is quantitatively illustrated in Fig. 5. The far-field distributions for a series of system sizes are shown in Fig. 5(a). Apparently, a large size is beneficial in realizing a narrowly diverged main-lobe and suppressing the amplitudes of the side-lobes. However, as shown in Fig. 5(b), the non-uniform power distribution is also very effective for suppressing the side-lobe. The side-lobe suppression ratio (the ratio of the peak intensities of the main lobe and the first side lobe) improves from 13.2 dB to 27.4 dB with a slight broadening (~0.01°) of the main lobe.

We further discuss the controllability of the proposed OPA. For simplicity, a zero phase *φ* = 0 is assigned initially, with a bias voltage of 0.85 V. Accordingly, the bias voltages of 0.4 V and 1.3 V give *φ* ≈ *π* and *φ* ≈ −*π*, respectively. In other words, a dynamic range of 900 mV in the bias voltage is large enough to support a phase shift from nearly −*π* to *π*. By tuning the bias voltages applied to the individual pixels, the phase distribution in the 2D array can be customized accordingly. As a result, a beam steering can be achieved.

**Figure 6:**
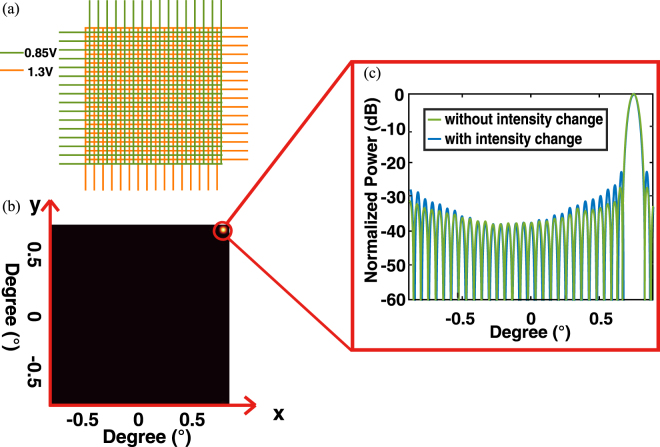
(**a**) Periodic bias voltage {0.85 V, 1.3 V} applied on each adjacent rows and columns. (**b**) Far-field pattern of beam steering. (**c**) Detailed angular power distribution in the x-axis cross section, with and without considering the change in intensity.

Figure 6(a) shows a periodic bias voltage {0.85 V, 1.3 V} applied on each adjacent rows and columns, which gives a phase shift of {0, *π*}. Due to the accompanying intensity modulation discussed above, extra insertion losses are induced, which are {0, 1.5 dB}, respectively. As these phase shifts are induced by on- and off-resonance with a maximum range of the optical resonance shifting, it also gives an upper bound of the accompanying intensity modulation. The far-field pattern of a given phase distribution is shown in Fig. 6(b), and its details along the x-axis cross section are shown in Fig. 6(c). It should be noted that, even with an intensity change of 1.5 dB, the far-field pattern maintains a good performance with a side-lobe suppression of over 25 dB. Beam steering towards other angles can be similarly realized by applying lower bias voltages on the pixels, thus, the phase shift and the intensity change are smaller. The presented example is the worst case of OPA beam steering operation.

### Tolerance to imperfections of fabrication

As the resonance wavelengths and the Q factors of the micro-rings are susceptible to fabrication faults and fluctuations, it is important to investigate the tolerance of the proposed OPA with respect to imperfections of fabrication. Such imperfections include stationary insertion losses (average value of random errors) and random fluctuated losses (standard deviation of random errors). The former stationary losses can be effectively compensated by tuning the coupling lengths of the individual pixels; however, certain residual errors can remain and contribute to the random fluctuated losses.

For an N × N square planar array, the root mean square (rms) of the pointing error with residual phase errors *δ* can be written as^44^:

5 $$rms=\frac{2\sqrt{3}\delta }{kd\,{\cos }(\psi )\,{(N-\mathrm{1)}}^{\mathrm{3/2}}}$$

For the presented 32 × 32 OPA, the maximum *ψ* is 1.78° and cos(*ψ*) within the full range of field are close to be constant. Therefore, for simplicity, *ψ* = 0 can be assumed, as it gives an appropriate approximation for the determination of the beam steering performance of the OPA in the full field of view.

**Figure 7:**
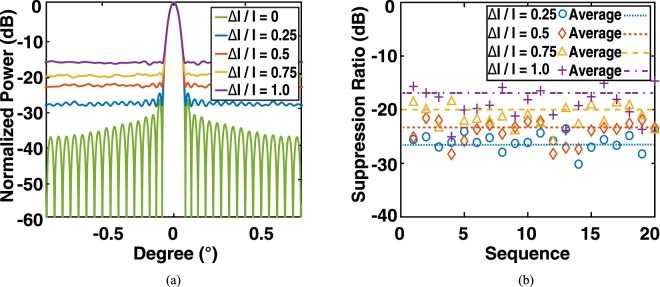
Monte Carlo simulations of the intensity error induced by the imperfections of fabrication, in which Δ*I*/*I* is assumed to be a normal distribution with standard deviations of 0, 0.25, 0.5, 0.75, 1.0, respectively. (**a**) Averaged far-field angular distribution. (**b**) Side-lobe suppression ratio (dB) in a sequence of simulations.

Here, we first perform an analysis of the OPA under an intensity error Δ*I*/*I* in which Δ*I* represents the magnitude of deviation of the emission power from its designed value at a given pixel, as shown in Fig. 7. A series of Monte Carlo simulations were performed for a normally distributed random Δ*I*/*I* with a given standard deviation. In particular, we first randomly generated a set of 32 × 32 resonance wavelengths obeying to a normal distribution *N*(*I*, Δ*I*) to present the micro-rings in the same chip, and then calculated the performance of the OPA accordingly. We repeated this procedure 20 times for a given *N*(*I*, Δ*I*) as Monte Carlo runs, in order to simulate a series of batches of samples. For the intensity error Δ*I*/*I* = 0.75 (3.4 dB), the side-lobe suppression ratio remains about 20 dB, which is still appropriate for an operational OPA. The results verify that the proposed OPA has a good tolerance to the variation of the power distribution. Figure 7 also provides an evaluation of the OPA performance under the accompanying intensity modulation. For a more sophisticated far-field pattern, the phase distribution on the 2D array is more “random” than periodic. As the upper bound of the intensity modulation is 1.5 dB and the inconsistency of efficiency of the grating coupler is 1.5 dB, it was expected that the side-lobe suppression ratio still remained above 20 dB under a 3-dB power variation in total.

One of the most important problems of the micro-ring arises from the variation of the resonance wavelengths and related *Q* factors due to the imperfections of fabrication. As the micro-ring modulator uses optical resonance to realize an effective phase shift, any variation of the resonances can directly result in fluctuations of the phase distribution on the OPA, thus, the far-field emission deviates from the designed pattern. To our knowledge, a state of the art fabrication technology can guarantee a device uniformity of <1% within a chip. For a series of micro-ring resonators placed in a 300-mm wafer, the resonance wavelength variations were measured as 0.15 nm in short distance (25 *μ*m) and 0.55 nm in large distance (1700 *μ*m), respectively^42,45^. Furthermore, 8 × 8 micro-ring switches were fabricated with a single optical input^46^.

**Figure 8:**
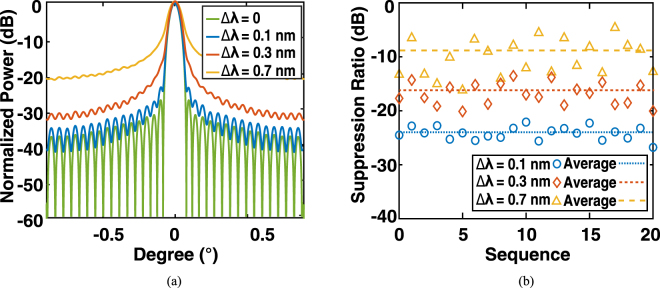
Monte Carlo simulations of the resonance wavelength error induced by the imperfections of fabrication, in which Δ*λ* is assumed to be a normal distribution with standard deviations of 0, 0.1 nm, 0.3 nm, and 0.7 nm, respectively. (**a**) Averaged far-field angular distribution. (**b**) Side-lobe suppression ratio (dB) in a sequence of simulations.

**Figure 9:**
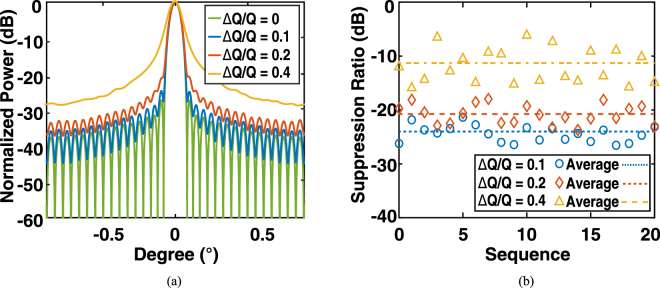
Monte Carlo simulations of the *Q* factor error induced by the imperfections of fabrication, in which Δ*Q*/*Q* is assumed to be a normal distribution with standard deviations of 0, 0.1, 0.2, and 0.4. (**a**) Averaged far-field angular distribution. (**b**) Side-lobe suppression ratio (dB) in a sequence of simulations.

**Figure 10:**
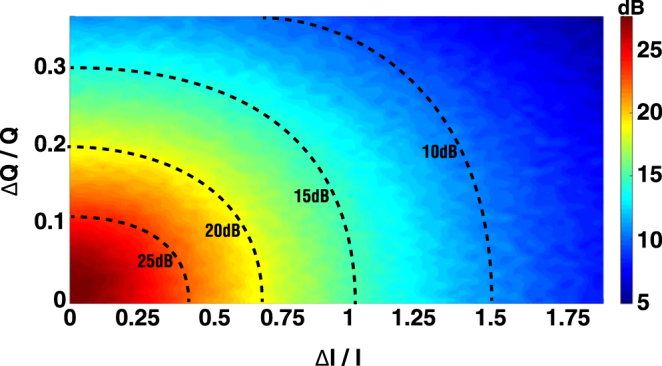
Side-lobe suppression ratio in the parameter space {Δ*I*/*I*, Δ*Q*/*Q*}.

Similarly, we present a series of Monte Carlo simulations to investigate the tolerance of the system with respect to the variation of the resonance wavelength and *Q* factor. The resonance wavelength *λ* is assumed to be randomly shifted by a standard deviation Δ*λ*, which statistically obeys a normal distribution *N*(*λ*, Δ*λ*). As shown in Fig. 8, considering the same main-lobe divergence, the side-lobe suppression ratio degrades to 16 dB and 9.5 dB, with Δ*λ* of 0.3 nm and 0.7 nm, respectively. Nevertheless, the variation of *Q* factors, denoted by Δ*Q*/*Q*, were investigated. As shown in Fig. 9, the side-lobe suppression ratio remains above 20 dB, and even the standard deviation of Δ*Q*/*Q* is as large as 0.2. The side-lobe suppression ratio in the parameter space {Δ*I*/*I*, Δ*Q*/*Q*} is shown in Fig. 10. This indicates that in order to maintain a suppression ratio higher than 20 dB, the variation of Δ*I*/*I* and Δ*Q*/*Q* have to be controlled under ~0.75 and ~0.2, respectively. According to the discussion above, such precision is feasible according to the state of the art precision of the fabrication process.

## Discussions

As mentioned above, the proposed OPA architecture can achieve a nearly full-range phase modulation (−*π* to *π*) with the modulation amplitude of about 900 mV. The device is quite robust against fluctuations in the fabrication. To maintain a side-lobe suppression ratio higher than 20 dB, its tolerance to the variation of the resonance wavelength is about 0.3 nm; the deviation of *Q* factor and power inequality are allowed as Δ*Q*/*Q* = 0.2 and Δ*I*/*I* = 0.75 (3.4 dB), respectively. Alternatively, to achieve a side-lobe suppression ratio higher than 10 dB, the variation of the resonance wavelength has to be below 0.7 nm; and the *Q* factor and power inequality have to satisfy Δ*Q*/*Q* = 0.4 and Δ*I*/*I* = 1.5 (8.5 dB). Therefore, for a system size of 32 × 32, the precision of controlling the Q factor and power inequality is feasible with respect to the fabrication process. The main challenge arises from the misalignment of the resonance wavelengths.

With state of the arts silicon photonic process, the resonance misalignment of micro-rings originates from the variations of the wafer thickness and lithography/etching precision. As the radius of a single silicon atom is as small as 110 pm, the variations in thickness are roughly in the range of ~0.1 nm in a distance of 300 *μ*m, and can be even larger over the whole wafer^47^. As a result, the resonance misalignment is determined as 0.7 nm in the proposed size of OPA. Nevertheless, it is reported^45^ that a resonance misalignment of 0.55 nm can be achieved in a 1700 *μ*m size by using a 0.13 *μ*m node with high-resolution optical lithography of 193 nm. This footprint is comparable with the proposed OPA (1.6 mm × 1.6 mm). As a result, we estimate that the resonance misalignment is about 0.7 nm, and it dominates the rising of the side-lobe, compared to the deviation of the Q factor and light intensity.

**Figure 11:**
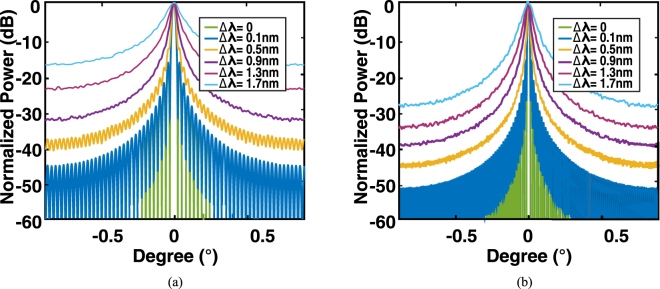
Monte Carlo simulations of the resonance wavelength error induced by the imperfections of fabrication, in which Δ*λ* is assumed to be a normal distribution with standard deviations of 0 to 1.7 nm, for system sizes of (**a**) 64 × 64; (**b**) 128 × 128.

It should be noted, that the tolerance to the resonance misalignment can be improved by increasing the size of the system, as shown in Fig. 11. Because the OPA is a Fourier optics system, the phase noise of the near field can be averaged out in the far field by the interference of the optical emission over all pixels. Although the increased size occupies a larger footprint, which results in a more severe resonance misalignment (up to 1.5 nm over the whole 200 mm wafer^48^), the tolerance to the resonance misalignment also remarkably improved. We believe that the resonance misalignment is not going to be a bottleneck of the system performance when increasing the size.

Moreover, the resonance wavelength is sensitive to the temperature. To maintain the fluctuation of the resonance wavelength under 0.7 nm, the temperature has be stabilized within 7 °C^42^. The temperature distribution across the entire array depends on the specific structural and thermal design of the device. Accurate control of the temperature at every individual EO pixel dramatically increases the complexity and cost of the electrical interconnection. We propose that an extra heating layer of titanium nitride (TiN) can be used to overspread on the layout, driven by the aluminum (Al) contacts, to maintain a constant temperature. The entire array can be divided into several regions with an independent heater in every region. Every region contains multiple rows and columns of EO pixels; therefore, the number of heating contacts can be reduced, while the temperature distribution can be controlled. Nevertheless, the wafer thickness is varying slowly across the wafer^47,48^. In particular, the uniformity of the thickness is appropriate in a small area, but slowly changing in different regions of the wafer. This suggests that the resonance misalignment due to the variation of thickness can also be partially compensated by a TiN heater.

The field of view mainly depends on the size of the EO pixel. In our model design, each pixel is in a 50 *μ*m × 50 *μ*m footprint with a micro-ring radius of 10 *μ*m, which gives a field of view of 1.78°. A larger field of view can be achieved by using a smaller ring radius, which results in a smaller doping region and a more compact EO pixel, while the phase efficiency is similar. A micro-ring modulator with a radius of 2.5 *μ*m^49^ has recently been reported. For our model OPA of 32 × 32 pixels, assuming a micro-ring radius of 2.5 *μ*m and a pixel size of 10 *μ*m the field of view can be maximally extended to 8.9°. Obviously, a smaller micro-ring radius also brings more challenges to the fabrication and integration. From a systematic point of view, we believe that the proposed pixel size is more practical for the demonstration of an operational OPA.

The proposed modularization can be easy extended to larger sizes without an exponential increase in complexity. Each EO pixel operates locally and independently, avoiding the layout problem of connecting the divided modulation and emission regions. Moreover, to support an *N* × *N* size of pixels, the number of light buses and control bus only linearly depends on *N*, which noticeably reduces complexity. However, the price to be paid is that the control bus only provides 2*N* degrees of freedom for phase manipulation, thus, it cannot generate an arbitrary phase pattern, such as in a holographic display, which requires *N* ^2^ degrees of freedom. Nevertheless, the proposed OPA is capable of realizing beam steering, which can be important for many applications, such as laser detection and ranging (LADAR), and free spatial communication.

Obviously, an independent and addressable control circuit can realize arbitrary phase patterns and it is also technically feasible. For instance, using a 3D integration technology, multiple metal contact layers can be vertically bonded on SOI chips^50^, which facilitates the layout of interconnections. The complexity of an addressable control circuit increases exponentially with the size of the system. As a result, a balance between complexity and functionality has to be found. The row/column control bus can be more practical to demonstrate an operational OPA for beam steering.

## Conclusion

In this work, a modularized architecture of a SOI-based OPA system using EO pixels has been presented. The pixel contains an evanescent coupler, a micro-ring phase shifter, and a grating optical antenna, on a compact size of 50 *μ*m × 50 *μ*m. A light bus and control bus scheme is used to distribute the optical powers and control signals to each pixel, and the complexity of its interconnections only linearly depends on the number of rows and columns. Moreover, we discuss the functionalities and characteristics of the key components, and provide useful details for the OPA design.

Furthermore, we present a model design of an OPA consisting of 32 × 32 EO pixels, which sets the width of the main lobe to 0.04° × 0.04° and the field of view as 1.78°. The micro-ring operates under an over-coupled condition to balance the modulation efficiency and the accompanying intensity modulation, which can potentially degrade the performance of the OPA. We validated that a change in peak intensity of 1.5 dB is acceptable. For the evaluation of the robustness of the proposed architecture, we present a series of Monte Carlo simulations on the variation of power inequities, resonance wavelengths, and Q factors. The discussion on the tolerance to the imperfections of fabrication shows that the proposed architecture is feasible regarding the state-of-the-art fabrication process. The performance of the main lobe width and field of view can be further optimized by larger system size and smaller pixel size. In addition, the modularized architecture can be readily extended to larger sizes without an exponential increase in complexity and cost of the interconnections.

The proposed architecture can be a promising choice to realize a large-scale OPA by integrating a large number of pixels into a single, compact, and low-cost chip. Owing to the speed of the EO modulation mechanism, such an OPA architecture has a significant advantage in terms of response time over a conventional OPA, based on low-speed MEMS and TO phase shifters, which can be important in several applications.

## Electronic supplementary material

Supplementary Information

## References

[1] McManamon PF (1996). Optical phased array technology. Proc. IEEE.

[2] Smit MK (1988). New focusing and dispersive planar component based on an optical phased array. Electron. Lett..

[3] Bogaerts W (2006). Compact Wavelength-Selective Functions in Silicon-on-Insulator Photonic Wires. IEEE J. Sel. Top. Quantum Electron..

[4] Yao J (2009). Microwave Photonics. J. Light. Technol..

[5] Trinh PD, Yegnanarayanan S, Coppinger F, Jalali B (1997). Silicon-on-insulator (SOI) phased-array wavelength multi/demultiplexer with extremely low-polarization sensitivity. IEEE Photonics Technol. Lett..

[6] Ng W (1991). The first demonstration of an optically steered microwave phased array antenna using true-time-delay. J. Light. Technol..

[7] Colburn S, Zhan A, Majumdar A (2017). Tunable metasurfaces via subwavelength phase shifters with uniform amplitude. Sci. Reports.

[8] Li J (2014). An Eight-Wavelength BH DFB Laser Array With Equivalent Phase Shifts for WDM Systems. IEEE Photonics Technol. Lett..

[9] Fryslie STM, Johnson MT, Choquette KD (2015). Coherence Tuning in Optically Coupled Phased Vertical Cavity Laser Arrays. IEEE J. Quantum Electron..

[10] Shay TM (2006). First experimental demonstration of self-synchronous phase locking of an optical array. Opt. Express, OE.

[11] Bochove EJ, Cheo PK, King GG (2003). Self-organization in a multicore fiber laser array. Opt. Lett., OL.

[12] Kapon E, Katz J, Yariv A (1984). Supermode analysis of phase-locked arrays of semiconductor lasers. Opt. Lett., OL.

[13] Riza NA (1994). Acousto-optic liquid-crystal analog beam former for phased-array antennas. Appl. Opt., AO.

[14] Cotter LK, Drabik TJ, Dillon RJ, Handschy MA (1990). Ferroelectric-liquid-crystal/silicon-integrated-circuit spatial light modulator. Opt. Lett., OL.

[15] Resler DP, Hobbs DS, Sharp RC, Friedman LJ, Dorschner TA (1996). High-efficiency liquid-crystal optical phased-array beam steering. Opt. Lett., OL.

[16] Riza NA (1992). Liquid crystal-based optical control of phased array antennas. J. Light. Technol..

[17] Wang B (2005). Stressed liquid-crystal optical phased array for fast tip-tilt wavefront correction. Appl. Opt., AO.

[18] Dolfi D, Michel-Gabriel F, Bann S, Huignard JP (1991). Two-dimensional optical architecture for time-delay beam forming in a phased-array antenna. Opt. Lett., OL.

[19] Yoo B-W (2014). A 32 × 32 optical phased array using polysilicon sub-wavelength high-contrast-grating mirrors. Opt. Express, OE.

[20] Bifano T (2011). Adaptive imaging: MEMS deformable mirrors. Nat Photon.

[21] Wang D (2013). Correction of image distortions in endoscopic optical coherence tomography based on two-axis scanning MEMS mirrors. Biomed. Opt. Express, BOE.

[22] Perruisseau-Carrier J, Skrivervik AK (2008). Monolithic MEMS-Based Reflectarray Cell Digitally Reconfigurable Over a 360° Phase Range. IEEE Antennas Wirel. Propag. Lett..

[23] Chan TK (2013). Optical beamsteering using an 8 × 8 MEMS phased array with closed-loop interferometric phase control. Opt. Express, OE.

[24] Shin J-D, Lee B-S, Kim B-G (2004). Optical true time-delay feeder for X-band phased array antennas composed of 2 × 2 optical MEMS switches and fiber delay lines. IEEE Photonics Technol. Lett..

[25] Sun J, Timurdogan E, Yaacobi A, Hosseini ES, Watts MR (2013). Large-scale nanophotonic phased array. Nat..

[26] Kwong D (2014). On-chip silicon optical phased array for two-dimensional beam steering. Opt. Lett., OL.

[27] Acoleyen KV, Komorowska K, Bogaerts W, Baets R (2011). One-Dimensional Off-Chip Beam Steering and Shaping Using Optical Phased Arrays on Silicon-on-Insulator. J. Light. Technol., JLT.

[28] Katayose, S., Hashizume, Y. & Itoh, M. 1 × 8 Silicon-silica hybrid thermo-optic switch with multi-chip configuration based on optical phased array. In *2015 20th Microoptics Conference* (*MOC*), 1–2, 10.1109/MOC.2015.7416395 (2015).

[29] Doylend, J. K. *et al*. Free-space beam steering in two dimensions using a silicon optical phased array. In *OFC*/*NFOEC*, 1–3 (2012).

[30] Robert, F. S. 2013 & Pm,. ScienceShot: Antenna Array Lights Up (2013).

[31] Tanemura, T., Langouche, L. & Nakano, Y. Strictly non-blocking 8 × 8 silicon photonic switch based on optical phased array. In *2015 European Conference on Optical Communication* (*ECOC*), 1–3, 10.1109/ECOC.2015.7341721 (2015).

[32] Hulme JC (2015). Fully integrated hybrid silicon two dimensional beam scanner. Opt. Express, OE.

[33] Reed GT, Mashanovich G, Gardes FY, Thomson DJ (2010). Silicon optical modulators. Nat Photon.

[34] Xu Q, Lipson M (2007). All-optical logic based on silicon micro-ring resonators. Opt. Express, OE.

[35] Yaacobi A (2014). Integrated phased array for wide-angle beam steering. Opt. Lett., OL.

[36] Sun J (2014). Large-Scale Silicon Photonic Circuits for Optical Phased Arrays. IEEE J. Sel. Top. Quantum Electron..

[37] Dolph CL (1946). A Current Distribution for Broadside Arrays Which Optimizes the Relationship between Beam Width and Side-Lobe Level. Proc. IRE.

[38] Meijerink A (2010). Novel Ring Resonator-Based Integrated Photonic Beamformer for Broadband Phased Array Receive Antennas Part I: Design and Performance Analysis. J. Light. Technol..

[39] Dong P (2009). Low V_pp_, ultralow-energy, compact, high-speed silicon electro-optic modulator. Opt. Express, OE.

[40] Xu Q, Manipatruni S, Schmidt B, Shakya J, Lipson M (2007). 12.5 Gbit/s carrier-injection-based silicon micro-ring silicon modulators. Opt. Express, OE.

[41] Soref R, Bennett B (1987). Electrooptical effects in silicon. IEEE J. Quantum Electron..

[42] Bogaerts W (2012). Silicon microring resonators. Laser & Photon. Rev..

[43] Halir R (2014). Recent Advances in Silicon Waveguide Devices Using Sub-Wavelength Gratings. IEEE J. Sel. Top. Quantum Electron..

[44] Carver K, Cooper W, Stutzman W (1973). Beam-pointing errors of planar-phased arrays. IEEE Transactions on Antennas Propag..

[45] Selvaraja SK, Bogaerts W, Dumon P, Thourhout DV, Baets R (2010). Subnanometer Linewidth Uniformity in Silicon Nanophotonic Waveguide Devices Using CMOS Fabrication Technology. IEEE J. Sel. Top. Quantum Electron..

[46] Nikolova D (2017). Modular architecture for fully non-blocking silicon photonic switch fabric. Microsystems & Nanoeng..

[47] Zortman WA, Trotter DC, Watts MR (2010). Silicon photonics manufacturing. Opt. Express, OE.

[48] Selvaraja, S. K. *et al*. Effect of device density on the uniformity of silicon nano-photonic waveguide devices. In *2009 IEEE LEOS Annual Meeting Conference Proceedings*, 311–312, 10.1109/LEOS.2009.5343250 (2009).

[49] Wu, R. *et al*. Large-Signal Model for Small-Size High-Speed Carrier-Injection Silicon Microring Modulator. In *Advanced Photonics 2016* (*IPR*, *NOMA*, *Sensors*, *Networks*, *SPPCom*, *SOF*) (*2016*), *paper IW1B*.*4*, IW1B.4, 10.1364/IPRSN.2016.IW1B.4 (Optical Society of America, 2016).

[50] Vivien, L. *et al*. European HELIOS project: Silicon photonic photodetector integration. In *2009 6th IEEE International Conference on Group IV Photonics*, 10–12, 10.1109/GROUP4.2009.5338308 (2009).

