# Supplementary material for: Blueprint for Large-Scale Silicon Optical Phased Array Using Electro-Optical Micro-Ring Pixels
Source: Sci Rep. 2017 Dec 18;7:17727. doi: 10.1038/s41598-017-18040-3 (PMC5735160; doi:10.1038/s41598-017-18040-3)
Supplement: Supplementary file 1 — Supplementary Information [file 41598_2017_18040_MOESM1_ESM.pdf]

Blueprint for Large-Scale Silicon Optical Phased Array Using Electro-Optical Micro-Ring Pixels

Che Zhao[1], **Chao Peng**[1,*], **and Weiwei Hu**[1,†]

[1]State Key Laboratory of Advanced Optical Communication Systems and Networks, School of Electronics Engineering and Computer Science, Peking University, Beijing, 100871, China
[*]pengchao@pku.edu.cn
[†]wwhu@pku.edu.cn

ABSTRACT

We propose a modularized architecture of a large-scale optical phased array (OPA) on a silicon on insulator (SOI) platform, using electro-optical (EO) pixels. Each pixel contains a directional coupler, a micro-ring phase shifter, and a grating optical antenna, on a compact configuration of area 50 μm $\times$ 50 μm, with optical and electrical interconnections. Moreover, we present an exemplary blueprint of an OPA consisting of 32 $\times$ 32 EO pixels, which sets the width of the main lobe as 0.04° $\times$ 0.04° and the field of view as 1.78°. By applying an over-coupled condition, the modulation efficiency and the accompanying intensity modulation are balanced, thus, the OPA performance is not severely degraded. The discussion on the fabrication tolerance shows that the proposed architecture is robust and feasible regarding the state-of-the-art fabrication process, and the performance of the main lobe width and field of view can be further optimized by a larger system size and smaller pixel size. Furthermore, the complexity of interconnections linearly depends on the number of rows and columns, making it highly scalable.

Appendix

The design and simulation parameters for the exemplary OPA design are presented as:

	Parameter	Value
**OPA system**	SOI wafer	220 nm Si / 2 μm SiO_2 box
	OPA system scale	32 $\times$ 32 = 1024
	OPA system footprint	1.6 mm $\times$ 1.6 mm
	OPA pixel footprint	50 μm $\times$ 50 μm
	Typical response time	<1 ns
	Incident wavelength λ	1552.4 nm
	Main lobe width (far-field)	0.04° $\times$ 0.04°
	Field of view	1.78°
	Side-lobe suppression ratio (SLSR)(with uniform power distribution)	13.2 dB
	Side-lobe suppression ratio (SLSR)(with non-uniform power distribution)	27.4 dB
**Directional coupler**	Coupling gap	150 nm
	Typical coupling length (efficiency)	3.1 μm (-10dB) and 7.4 μm (-3dB)
	Relative phase shifts	<0.05 π
**EO micro-ring phase shifter**	Waveguide rib width	450 nm
	Waveguide rib height	130 nm
	Micro-ring radius	10 μm
	Material, P^{++} doping density	Boron, 10^{20}/cm^3
	Material, N^{++} doping density	Phosphorus, 10^{20}/cm^3
	Bias voltage	0.4 V to 1.3 V
	Modulation efficiency on effective refractive index change	5 $\times$ 10^{-3} / 100 mV.
	Peak-to-peak intensity variation on full 2 π phase change	1.5 dB
	Q factor	$\sim$2760
	Self-coupling coefficient r	0.85
	Amplitude transmission coefficient a	0.96
**Arc-shaped grating coupler**	Length	3.5 μm
	Width	6.4 μm
	Period of the two adjacent	0.68 μm
	Insertion loss	4.5 dB to 6 dB
**Tolerance and error analysis**	Resonance misalignment standard deviation ($\Delta\lambda$) (20 dB SLSR)	0.3 nm
	Resonance misalignment standard deviation ($\Delta\lambda$) (10 dB SLSR)	0.7 nm
	Resonance misalignment max-min range (at $\Delta\lambda$=0.7 nm for 32 $\times$ 32 rings)	$\sim$1.2 nm to $\sim$0.2 nm
	Intensity standard deviation ($\Delta I/I$) (20 dB SLSR)	0.75
	Intensity standard deviation ($\Delta I/I$) (10 dB SLSR)	1.50
	Q factor standard deviation ($\Delta Q/Q$) (20 dB SLSR)	0.2
	Q factor standard deviation ($\Delta Q/Q$) (10 dB SLSR)	0.4
	Monte-carlo runs	20
